# Supplementary material for: Timing of geological events in the lunar highlands recorded in shocked zircon-bearing clasts from Apollo 16
Source: R Soc Open Sci. 2020 Jun 3;7(6):200236. doi: 10.1098/rsos.200236 (PMC7353986; doi:10.1098/rsos.200236)
Supplement: Electronic Supplementary Materials - Figures and Note 1 [file rsos200236supp1.docx]

**Electronic Supplementary Materials**

Timing of geological events in the lunar highlands recorded in shocked zircon bearing clasts from Apollo 16

K. H. Joy^1^, J. F. Snape^2^, A. A. Nemchin^2,3^, R. Tartèse^1^, D. M. Martin^4^, M. J. Whitehouse^2^, V. Vishnyakov^5^, J. F. Pernet-Fisher^1^, and D.A. Kring^6^.


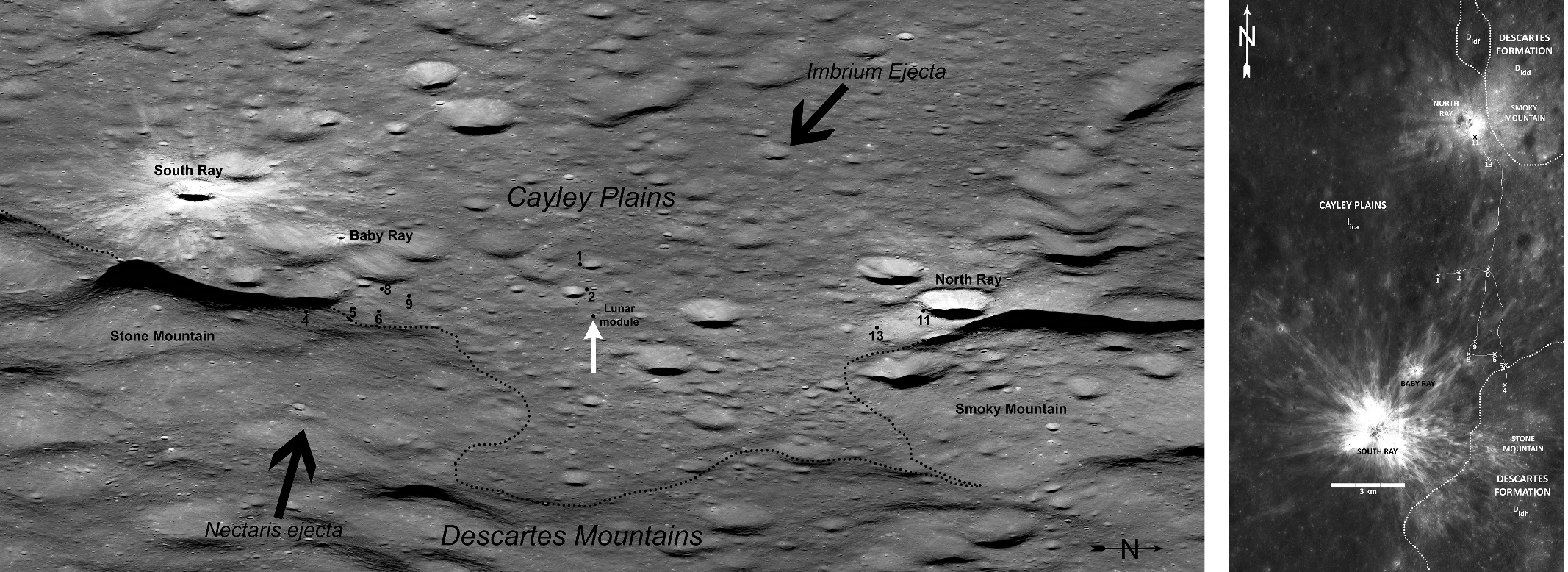


Figure S1. Left: Oblique view of the Apollo 16 landing site showing surface geomorphological content. The lunar module landing location, sample collection stations (note that sample 65745 was picked up from Station 5) and local geological boundaries are indicated. Note that North is to the right of the image frame and that the distance from South Ray to North Ray crater is 10.4 km. The image has been modified from LROC NAC frame M192817484LR (see http://lroc.sese.asu.edu/posts/529). Right: Overhead view of the Apollo 16 landing site. Apollo 16 mission traverse route and sample stations with collection locations of the Apollo 16 regolith breccias discussed in this study. Data are overlain on montage of LROC NAC frames M106777343RE and M106777343LE with original spatial resolution of ~1.10 m/ pixel and taken at high-sun at an incidence angle of 28º. Geological boundary between the Cayley Plains Formation and the Descartes Formation (Hodges, 1972) is shown as white dashes. Smoky Mountain to the North is part of the Descartes D_idd_ domed unit with a small D_idf_ furrowed unit. Stone Mountain to the south is part of the Descartes D_idh_ hilly unit. Image modified after Joy et al. (2011)


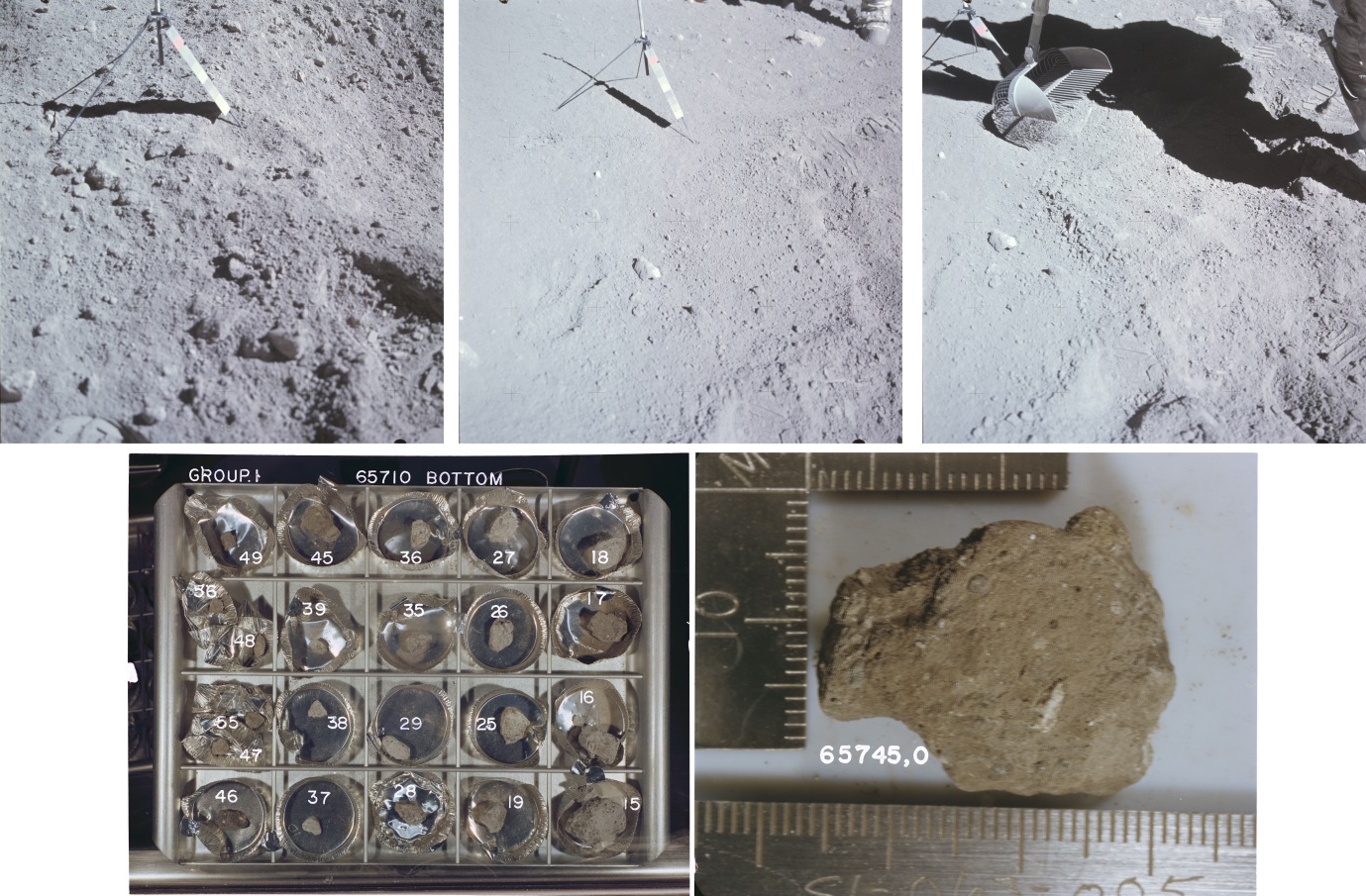


Figure S2. Upper frames: Pre (left and centre) and post (right) locations of 65745 and other rake samples collection site at station 5. Images: NASA Hasselblad frames AS16-107-17493, AS16-107-17497 and AS16-107-17498 (with thanks to the Apollo Lunar Surface Journal). Lower left: rake breccias collected at Station 5 (sample 65745 is in the top row, second from left labelled 45). Lower Right: Hand specimen of 2.6 × 2.2 × 1.2 cm sized 65745 showing brownish colour. Images: NASA curatorial images S72-43199 and S72-47692.


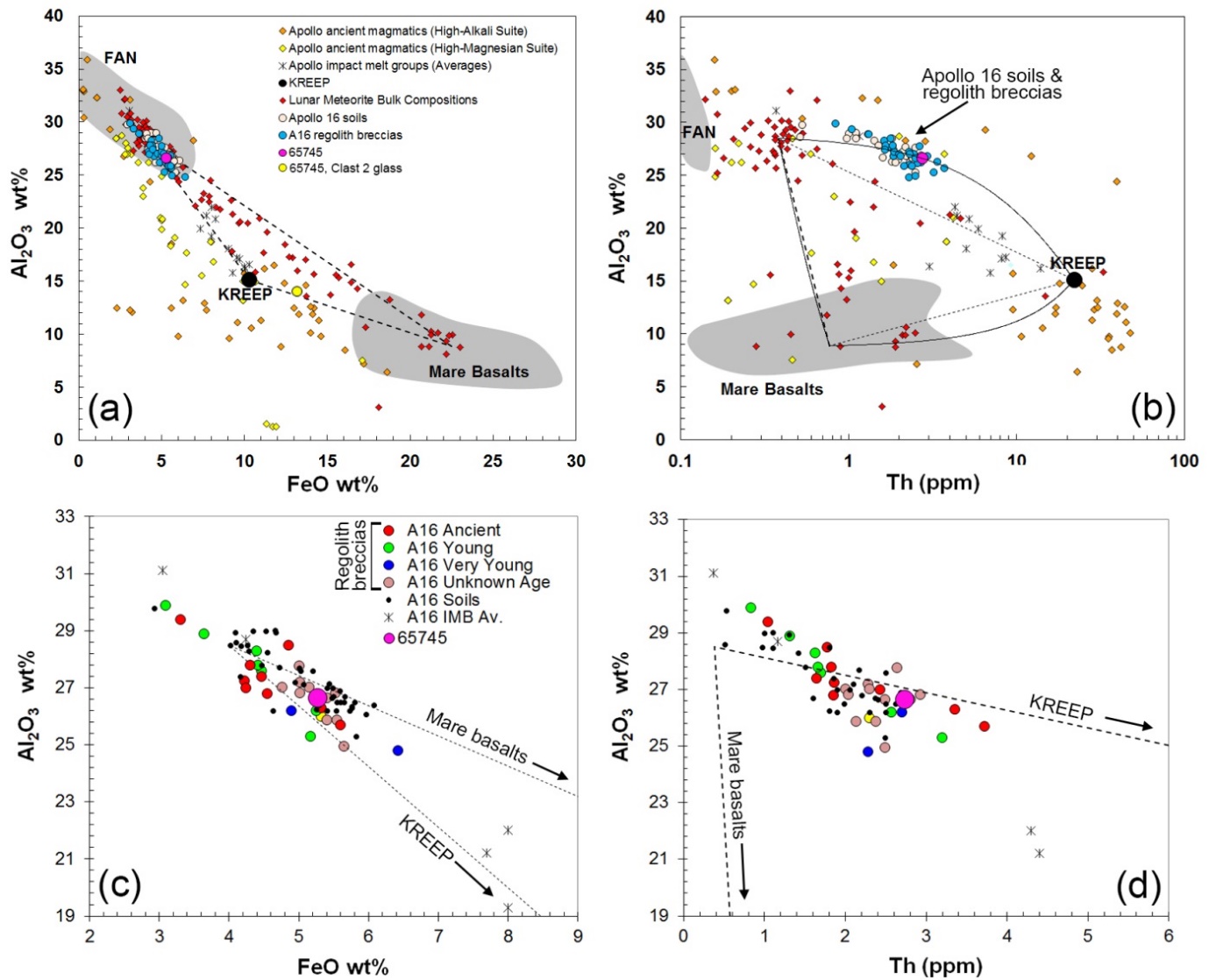


Figure S3. Lunar geochemical context of regolith breccia 65745. (a) and (c) bulk rock Al_2_O_3_ vs. FeO (in wt%), and (b) and (d) Al_2_O_3_ (in wt%) vs. Th (in ppm). Sample 65745 is a feldspathic regolith breccia of unknown formation age, however, as it is soil-like it is likely comparable to the very young age group consolidated within the last 1 Ga or so.


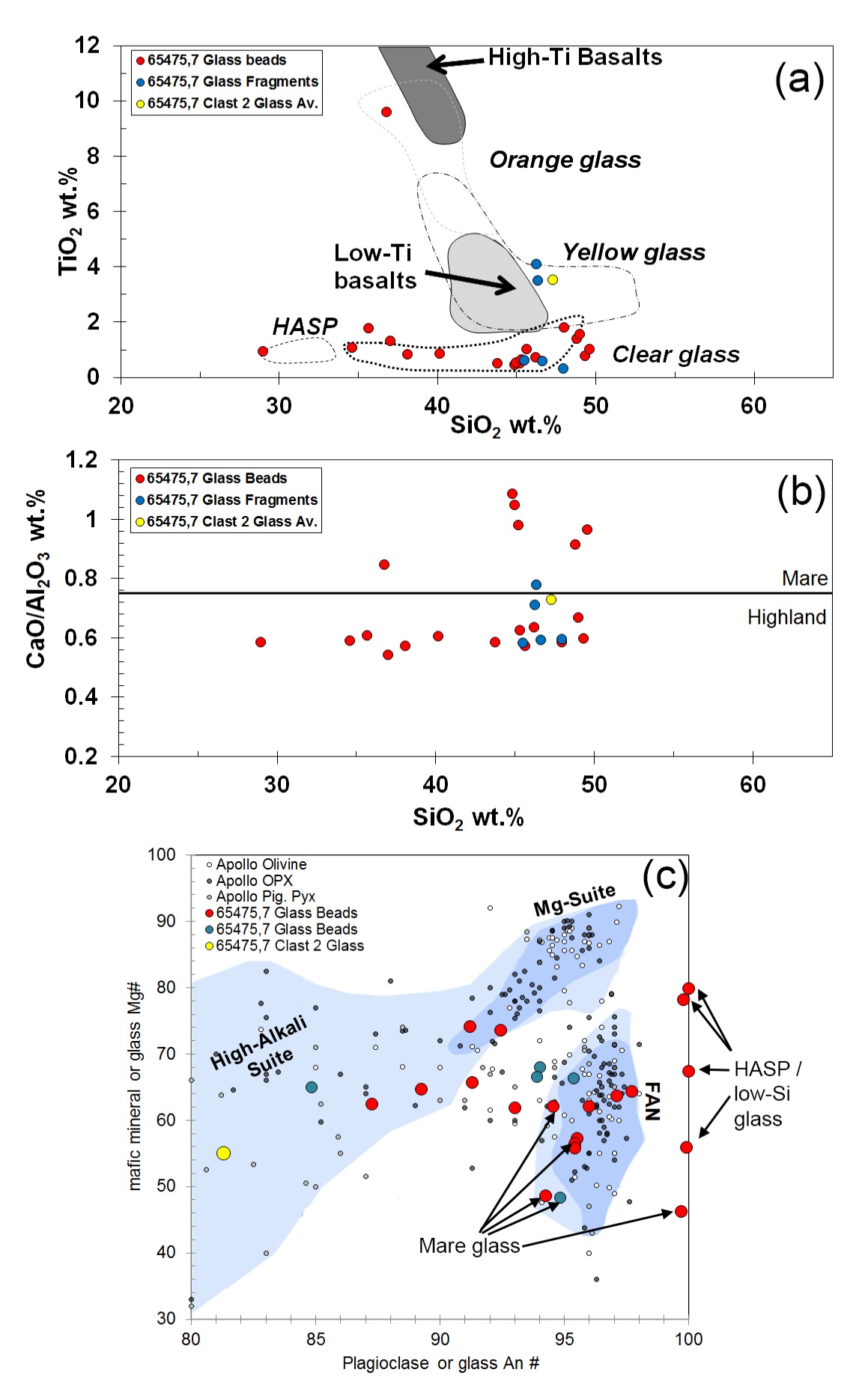


Figure S4. Composition of glass particles (volcanic glass beads, impact melt beads, glass fragments, and agglutinitic glass; Table S2) within the matrix of 65745,7 compared with the average composition of the devitrified glass in the Clast 2 impact melt breccia (Table S1).

Table S5: NanoSIMS detector configuration and analysis time.

| Acquisition | Field | Dwell time (μs/px) | EM#1 | EM#2 | EM#3 | EM#4 | EM#5 | EM#6 | EM#7 |
| --- | --- | --- | --- | --- | --- | --- | --- | --- | --- |
| #1 | B1 | 2000 |  |  |  |  |  | 203.5 |  |
|  | B2 | 5000 | ^28^Si | ^48^Ti | ^56^Fe | ^89^Y | ^94^Zr | ^204^Pb | ^208^Pb |
|  | B3 | 5000 |  |  |  |  |  | ^206^Pb |  |
|  | B4 | 8000 |  |  |  |  |  | ^207^Pb |  |
| #2 | B1 | 4000 | ^28^Si | ^32^SO_2_ |  |  |  | ^232^ThO | ^238^UO |

**Supplementary Note 1.**

Additional details about the Cameca SX100 electron microprobe zircon data collection process, including details of peak setup, crystals, standards, and peak position and backgrounds.

Quantitative Analysis Declaration

**Element Peak Crystal Spectrometer Standard Typ. det. limit.***

Si Ka TAP Sp5 wol 0.02

Mg Ka TAP Sp5 per 0.02

K Ka PET Sp4 ksp 0.03

P Ka TAP Sp5 apt **not acquired

Ti Ka PET Sp4 rut 0.03

Sc Ka LPET Sp3 Scandium 0.007

Ce La LLIF Sp1 Ce2gl 0.1

Fe Ka LIF Sp2 fay 0.1

Lu La LIF Sp2 Lu2gl 0.5

Yb La LIF Sp2 Yb2glass 0.35

Cl Ka LPET Sp3 hal 0.03

Al Ka TAP Sp5 cor 0.01

Ca Ka PET Sp4 wol 0.03

Hf Ma TAP Sp5 Hafnium3 0.06

Ba La LPET Sp3 bagl **not acquired

Y La PET Sp4 Y2gl 0.1

Zr La PET Sp4 zr 0.3

Th Ma LPET Sp3 Th2gl 0.07

Pb Ma LPET Sp3 PbCrO4 0.08

U Ma LPET Sp3 U 0.1

Nd Lb LLIF Sp1 Nd2gl 0.18

Eu Lb LLIF Sp1 Eu2gl **not acquired

Sm Lb LLIF Sp1 Sm2gl 0.22

La La LLIF Sp1 La2gl 0.1

* in unknown zircon in element wt% (note those in Table S3 are reported in oxide wt%)

**not acquired on zircon run on 04/02/2014

Label:misc_Zirc

Date:Tue Mar 4 08:22:51 2014

Type:Geo

Take Off Angle:40.

Iteration Limit:0.000

Condition #1 15.0 kV 20.0 nA

Sp1 LLIF

Ce La Shift:0 Valence:3

Time(sec):30 Bkg(sec):0.0

(+)Bkg:650 (-)Bkg:-550 Slope:0.000

Standard:Ce2gl Esti 3 Sig.D.L.(ppm): ?

Bias(V):1855 Gain(*):417 DeadTime(us):3

Baseline:2200 Window:1300 Mode:Pha Integral

SineTheta:63632 I(C/s/nA):15.818 Date:03/Mar/14

Nd Lb Shift:0 Valence:3

Time(sec):30 Bkg(sec):0.0

(+)Bkg:300 (-)Bkg:-350 Slope:0.000

Standard:Nd2gl Esti 3 Sig.D.L.(ppm): ?

Bias(V):1855 Gain(*):417 DeadTime(us):3

Baseline:2200 Window:1300 Mode:Pha Integral

SineTheta:53829 I(C/s/nA):12.020 Date:03/Mar/14

Eu Lb Shift:0 Valence:2

Time(sec):30 Bkg(sec):0.0

(+)Bkg:400 (-)Bkg:-400 Slope:0.000

Standard:Eu2gl Esti 3 Sig.D.L.(ppm): ?

Bias(V):1855 Gain(*):417 DeadTime(us):3

Baseline:2200 Window:1300 Mode:Pha Integral

SineTheta:47693 I(C/s/nA):12.109 Date:03/Mar/14

Sm Lb Shift:0 Valence:2

Time(sec):30 Bkg(sec):0.0

(+)Bkg:500 (-)Bkg:-500 Slope:0.000

Standard:Sm2gl Esti 3 Sig.D.L.(ppm): ?

Bias(V):1820 Gain(*):374 DeadTime(us):3

Baseline:1500 Window:1000 Mode:Pha Integral

SineTheta:49635 I(C/s/nA):11.127 Date:03/Mar/14

La La Shift:0 Valence:3

Time(sec):30 Bkg(sec):0.0

(+)Bkg:700 (-)Bkg:-500 Slope:0.000

Standard:La2gl Esti 3 Sig.D.L.(ppm): ?

Bias(V):1855 Gain(*):417 DeadTime(us):3

Baseline:2200 Window:1300 Mode:Pha Integral

SineTheta:66221 I(C/s/nA):14.735 Date:03/Mar/14

Sp2 LIF

Fe Ka Shift:0 Valence:2

Time(sec):30 Bkg(sec):0.0

(+)Bkg:500 (-)Bkg:-500 Slope:0.000

Standard:fay Esti 3 Sig.D.L.(ppm): ?

Bias(V):1305 Gain(*):421 DeadTime(us):3

Baseline:560 Window:1771 Mode:Pha Integral

SineTheta:48084 I(C/s/nA):62.050 Date:03/Mar/14

Lu La Shift:0 Valence:3

Time(sec):30 Bkg(sec):0.0

(+)Bkg:400 (-)Bkg:-300 Slope:0.000

Standard:Lu2gl Esti 3 Sig.D.L.(ppm): ?

Bias(V):1305 Gain(*):421 DeadTime(us):3

Baseline:560 Window:1771 Mode:Pha Integral

SineTheta:40182 I(C/s/nA):2.580 Date:03/Mar/14

Yb La Shift:0 Valence:3

Time(sec):30 Bkg(sec):0.0

(+)Bkg:450 (-)Bkg:-300 Slope:0.000

Standard:Yb2glass Esti 3 Sig.D.L.(ppm): ?

Bias(V):1311 Gain(*):425 DeadTime(us):3

Baseline:560 Window:1771 Mode:Pha Integral

SineTheta:41515 I(C/s/nA):3.204 Date:03/Mar/14

Sp3 LPET

Sc Ka Shift:0 Valence:3

Time(sec):30 Bkg(sec):0.0

(+)Bkg:400 (-)Bkg:-400 Slope:0.000

Standard:Scandium Esti 3 Sig.D.L.(ppm): ?

Bias(V):1833 Gain(*):818 DeadTime(us):3

Baseline:1750 Window:1250 Mode:Pha Integral

SineTheta:34638 I(C/s/nA):3016.201 Date:03/Mar/14

Cl Ka Shift:0 Valence:-1

Time(sec):10 Bkg(sec):0.0

(+)Bkg:500 (-)Bkg:-500 Slope:0.000

Standard:hal Esti 3 Sig.D.L.(ppm): ?

Bias(V):1833 Gain(*):818 DeadTime(us):3

Baseline:1750 Window:1250 Mode:Pha Integral

SineTheta:54060 I(C/s/nA):906.005 Date:03/Mar/14

Ba La Shift:0 Valence:2

Time(sec):30 Bkg(sec):0.0

(+)Bkg:300 (-)Bkg:-300 Slope:0.000

Standard:bagl Esti 3 Sig.D.L.(ppm): ?

Bias(V):1823 Gain(*):798 DeadTime(us):3

Baseline:1000 Window:900 Mode:Pha Integral

SineTheta:31716 I(C/s/nA):89.946 Date:03/Mar/14

Th Ma Shift:0 Valence:4

Time(sec):30 Bkg(sec):0.0

(+)Bkg:300 (-)Bkg:-300 Slope:0.000

Standard:Th2gl Esti 3 Sig.D.L.(ppm): ?

Bias(V):1844 Gain(*):865 DeadTime(us):3

Baseline:1000 Window:900 Mode:Pha Integral

SineTheta:47289 I(C/s/nA):16.242 Date:03/Mar/14

Pb Ma Shift:0 Valence:2

Time(sec):30 Bkg(sec):0.0

(+)Bkg:500 (-)Bkg:-500 Slope:0.000

Standard:PbCrO4 Esti 3 Sig.D.L.(ppm): ?

Bias(V):1831 Gain(*):818 DeadTime(us):3

Baseline:1000 Window:900 Mode:Pha Integral

SineTheta:60420 I(C/s/nA):163.473 Date:03/Mar/14

U Ma Shift:0 Valence:3

Time(sec):30 Bkg(sec):0.0

(+)Bkg:250 (-)Bkg:-100 Slope:0.000

Standard:U Esti 3 Sig.D.L.(ppm): ?

Bias(V):1823 Gain(*):798 DeadTime(us):3

Baseline:1000 Window:900 Mode:Pha Integral

SineTheta:44672 I(C/s/nA):365.460 Date:03/Mar/14

Sp4 PET

K Ka Shift:0 Valence:1

Time(sec):20 Bkg(sec):10.0

(+)Bkg:250 (-)Bkg:-350 Slope:0.000

Standard:ksp Esti 3 Sig.D.L.(ppm): ?

Bias(V):1328 Gain(*):1003 DeadTime(us):3

Baseline:741 Window:2759 Mode:Pha Integral

SineTheta:42761 I(C/s/nA):65.239 Date:03/Mar/14

Ti Ka Shift:0 Valence:4

Time(sec):20 Bkg(sec):10.0

(+)Bkg:250 (-)Bkg:-350 Slope:0.000

Standard:rut Esti 3 Sig.D.L.(ppm): ?

Bias(V):1319 Gain(*):987 DeadTime(us):3

Baseline:741 Window:3559 Mode:Pha Integral

SineTheta:31411 I(C/s/nA):366.089 Date:03/Mar/14

Ca Ka Shift:0 Valence:2

Time(sec):20 Bkg(sec):10.0

(+)Bkg:400 (-)Bkg:-400 Slope:0.000

Standard:wol Esti 3 Sig.D.L.(ppm): ?

Bias(V):1328 Gain(*):1003 DeadTime(us):3

Baseline:741 Window:2759 Mode:Pha Integral

SineTheta:38387 I(C/s/nA):194.689 Date:03/Mar/14

Y La Shift:0 Valence:3

Time(sec):30 Bkg(sec):0.0

(+)Bkg:500 (-)Bkg:-500 Slope:0.000

Standard:Y2gl Esti 3 Sig.D.L.(ppm): ?

Bias(V):1331 Gain(*):1013 DeadTime(us):3

Baseline:1650 Window:1150 Mode:Pha Integral

SineTheta:73731 I(C/s/nA):5.563 Date:03/Mar/14

Zr La Shift:0 Valence:4

Time(sec):30 Bkg(sec):0.0

(+)Bkg:500 (-)Bkg:-500 Slope:0.000

Standard:zr Esti 3 Sig.D.L.(ppm): ?

Bias(V):1331 Gain(*):1013 DeadTime(us):3

Baseline:1650 Window:1150 Mode:Pha Integral

SineTheta:69401 I(C/s/nA):86.790 Date:03/Mar/14

Sp5 TAP

Si Ka Shift:0 Valence:4

Time(sec):30 Bkg(sec):0.0

(+)Bkg:300 (-)Bkg:-400 Slope:0.000

Standard:wol Esti 3 Sig.D.L.(ppm): ?

Bias(V):1290 Gain(*):2543 DeadTime(us):3

Baseline:560 Window:2600 Mode:Pha Integral

SineTheta:27737 I(C/s/nA):441.175 Date:03/Mar/14

Mg Ka Shift:0 Valence:2

Time(sec):20 Bkg(sec):10.0

(+)Bkg:350 (-)Bkg:-550 Slope:0.000

Standard:per Esti 3 Sig.D.L.(ppm): ?

Bias(V):1291 Gain(*):2597 DeadTime(us):3

Baseline:560 Window:2600 Mode:Pha Integral

SineTheta:38477 I(C/s/nA):817.293 Date:03/Mar/14

P Ka Shift:0 Valence:5

Time(sec):20 Bkg(sec):10.0

(+)Bkg:300 (-)Bkg:-350 Slope:0.000

Standard:apt Esti 3 Sig.D.L.(ppm): ?

Bias(V):1282 Gain(*):2516 DeadTime(us):3

Baseline:560 Window:2600 Mode:Pha Integral

SineTheta:23982 I(C/s/nA):294.579 Date:03/Mar/14

Al Ka Shift:0 Valence:3

Time(sec):20 Bkg(sec):10.0

(+)Bkg:500 (-)Bkg:-500 Slope:0.000

Standard:cor Esti 3 Sig.D.L.(ppm): ?

Bias(V):1290 Gain(*):2543 DeadTime(us):3

Baseline:560 Window:2600 Mode:Pha Integral

SineTheta:32448 I(C/s/nA):928.223 Date:03/Mar/14

Hf Ma Shift:0 Valence:4

Time(sec):30 Bkg(sec):0.0

(+)Bkg:500 (-)Bkg:-500 Slope:0.000

Standard:Hafnium3 Esti 3 Sig.D.L.(ppm): ?

Bias(V):1296 Gain(*):2556 DeadTime(us):3

Baseline:560 Window:2600 Mode:Pha Integral

SineTheta:29320 I(C/s/nA):601.675 Date:03/Mar/14

Not analyzed :O

Number of Oxygens = 24

Number of H2O = 0
